# Approaches to Manipulating the Dimensionality and Physicochemical Properties of Common Cellular Scaffolds

**DOI:** 10.3390/ijms12128596

**Published:** 2011-11-29

**Authors:** Saumendra Bajpai, Na Young Kim, Cynthia A. Reinhart-King

**Affiliations:** Department of Biomedical Engineering, Cornell University, 302 Weill Hall, Ithaca, NY 14853, USA; E-Mails: cal_baj@yahoo.com (S.B.); nk326@cornell.edu (N.Y.K.)

**Keywords:** substrate, scaffold, collagen, gel, cross-linker, stiffness, mechanotransduction

## Abstract

A major hurdle in studying biological systems and administering effective tissue engineered therapies is the lack of suitable cell culture models that replicate the dynamic nature of cell-microenvironment interactions. Advances in the field of surface chemistry and polymer science have allowed researchers to develop novel methodologies to manipulate materials to be extrinsically tunable. Usage of such materials in modeling tissues *in vitro* has offered valuable insights into numerous cellular processes including motility, invasion, and alterations in cell morphology. Here, we discuss novel techniques devised to more closely mimic cell-tissue interactions and to study cell response to distinct physico-chemical changes in biomaterials, with an emphasis on the manipulation of collagen scaffolds. The benefits and pitfalls associated with using collagen are discussed in the context of strategies proposed to control the engineered microenvironment. Tunable systems such as these offer the ability to alter individual features of the microenvironment *in vitro*, with the promise that the molecular basis of mechanotransduction *in vivo* may be laid out in future.

## 1. Introduction

**Figure 1:**
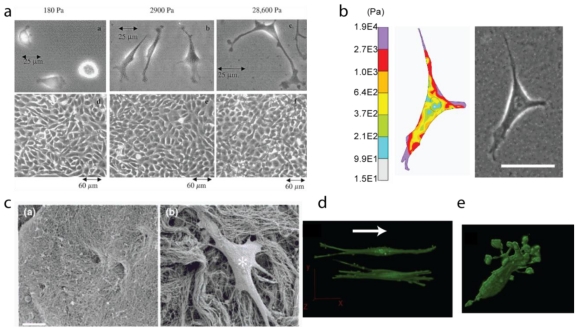
Cellular responses to change in extracellular cues. (**a**) Variation of cell morphology with changing substrate stiffness. Bovine aortic endothelial cells (BAEC) plated on collagen coated 2D polyacrylamide (PA) substrates with varying stiffness exhibit varying cell-morphology [9]. Reprinted from [9] with permission from John Wiley and Sons; (**b**) The spatial variation of stress distribution as measured for a BAEC [10,11] adhered to polyacrylamide gel (E = 2.5 kPa). Image courtesy of Joseph P. Califano. (**c**) The nature of gradients exhibited by *in vivo* interfaces. *In vivo* architecture of the basement membrane **c**(**a**) and the dermis **c**(**b**), with a fibroblast sprawled in quasi-3D architecture. The tissue architecture changes from **c**(**a**) to **c**(**b**) over a few tens of microns [12]. Reprinted from [12] with permission from Elsevier; (**d**) and (**e**) show the effect of fiber alignment on cell morphology for cells inside 3D collagen matrices with and without imposed alignment respectively. Arrow marks the direction of fiber alignment that induces nearly similar alignment of the cells [13,14]. Reprinted from [14] with permission from Elsevier.

Tissue structure is one result of the evolutionary pressures imposed to satisfy some of the most complex needs for heat, mass, and fluid transfer within the human body. Efficient transfer of nutrients and factors that govern induction of time-critical pathways and action-at-a-distance communication between cells within a tissue and those between dissimilar tissues are principal determinants of tissue functionality and pathogenic states. Due to the complexities involved in studying live tissues in real-time, most research on cell behavior has traditionally focused on two-dimensional (2D) cell culture [1] and more recently, on culturing cells within three-dimensional (3D) matrices [2–4] (Figure 1). While the general focus in recent literature has been moving from simple, non-representative systems to more complex systems mimicking real tissues more closely, discrepancies between *in-vitro* observations and *in-vivo* behavior continue to be numerous.

There are several key differences between the cell behaviors described in microenvironments created by various cell culture models and the *in vivo* conditions these models aim to replicate [5–8]. In this review, we examine some of the recent advances in the modulation of cellular microenvironment aimed at mimicking real tissues more closely. As a general trend, the more current focus in this field has been on designing systems with biomechanical characteristics that can be tuned and controlled without inducing cellular toxicity/incompatibility.

## 2. Engineering the Two Dimensional Landscape

Traditionally, cells harvested from tissues (and subsequently used as primary cells or as immortalized cell lines) have been cultured on 2D surfaces that have been pretreated with adhesion-favoring ligands. Historically, much of what we know from the literature within the field of cell biology is based on information derived from studies performed using cells plated on polystyrene dishes. For instance, most of the cell migration literature is composed of studies of cells on polystyrene dishes, where for example, wound healing is simulated by scratching a monolayer with a pipette tip. Likewise, much attention has been paid to the role of focal adhesions on the basal surface of cells cultured on plastic dishes and their role in cell motility [13]. Additionally, the role played by phosphorylation of variation signaling proteins in governing cell motility was largely elucidated by cells cultured on 2D hard surfaces [15–18]. However, there remains a debate as to whether focal adhesions occur in cells embedded in 3D microenvironments [19,20]. The differences between 2D substrates and the 3D *in vivo* environment are becoming increasingly more important to the biological community as evidence mounts indicating that the two conditions (2D *versus* 3D) can cause entirely different cell responses.

While it is known that 2D environments do not fully recapitulate what cells experience *in vivo*, they most often continue to be the platform of choice due to their ease of use and availability. In recent years, researchers have sought to re-engineer 2D substrates to more closely mimic the critical features present in the 3D, *in vivo* microenvironment.

One of the critical differences between traditional culture and *in vivo* tissue conditions is the dissimilarity in cell and matrix polarity cues that are present. Culture in a petri dish imposes apical-basal polarizing cues otherwise not found in the *in vivo* microenvironment [21]. The inherent baso-apical polarity induced by 2D culture of fibroblasts cells on polystyrene dishes is a classic case in point where the culture condition imposes an artificial polarity, resulting in altered cell behavior. While significant information about the morphology and motility of these cells is reported using 2D cultures [21], many of these results are not recapitulated in real tissues. For instance, the faster modes of cell motility discovered with mammalian carcinoma cells invading inside tissues (with speeds up to 3 μm/min [22]) have not been reported for cells moving on 2D culture plastic or inside 3D collagen gels. The differences in behaviors exhibited by cells on polystyrene as compared to those *in vivo* are likely due to the differences between the polarizing cues found in each condition.

Numerous 2D approaches have been proposed to control polarity and study the correlation between cell polarization and cell behavior. To confine and direct cell polarization, soft lithography has been an important tool in the array of techniques proposed to design tunable biomaterials [23]. Methods to confine the distribution of extracellular matrix (ECM) ligands and non-adhesive molecules using microcontact printing have enabled the creation of patterns that can prescribe the polarization of cells cultured on 2D surfaces. These systems have been optimized to pattern shapes of extracellular matrix protein at length scales that are either far smaller or far larger than that of a single cell [24,25]. Engineered polarization of cell morphology has enabled the investigation of the interplay between cell polarity and cell motility [26,27]. Similarly, soft lithography has facilitated the investigation of the critical role played by cell shape and cell-cell contact in cell motility and wound healing [28].

One of the most significant differences between traditional culture conditions and *in vivo* systems is the striking dissimilarity in their mechanical properties. Mammalian tissues exhibit a vast range of stiffness: from a few Pascals to several hundred kiloPascals (Table 1). To expose cells to matrices mimicking the mechanical properties that reflect the conditions that are native to the cell type under investigation, several different platforms have been proposed where each methodology exhibits varying degrees of tunability. Foremost amongst them are polyacrylamide-based substrates, wherein cells are plated on polyacrylamide derivatized with various ECM proteins [10,11,29–31] (Figure 1(a)). The degree of crosslinking induced at the time of polymerization provides tight control over the stiffness of the resulting substrate. In addition to the pivotal role the polyacrylamide platform has played in understanding how matrix mechanics can alter cell behavior, the advent of polyacrylamide gels has also enabled the development of Traction Force Microscopy, a computational method which calculates the traction stresses exerted by cells on their substrate [10,32] (Figure 1(b)).

In addition to the use of polyacrylamide substrates, other methods have also been developed to investigate the effects of matrix stiffening on cell behavior. For instance, several approaches have been described to stiffen collagen. While collagen can be stiffened by increasing its density, the stiffness can also be manipulated independently of density through crosslinking using fixatives such as glutaraldehyde or non-enzymatically using glycation [42,43]. The benefit of using collagen, in comparison to most other polymer systems, is that it occurs naturally. However, the range of stiffnesses that can be achieved is limited.

As a step towards moving from 2D substrates to 3D microenvironments, pseudo-3D systems have been created. Here, cells are plated between two orthogonal 2D surfaces coated with collagen I [44,45]. In this system, cells spread over a typical 2D surface, but the cells can also bind to the substrate on their apical side. The advantage of these pseudo-3D environments over fully 3D environments is that they are easier to image and manipulate. It is possible that results obtained from these pseudo-3D systems can shed some light on the behavior of cells in tissues, especially near tissue boundaries that can exhibit both a stiffness gradient and a topographical change in the microenvironment.

To create 2D substrates containing gradients of stiffness, various microfluidic systems, where the mixing of pre-polymerized matrices can be controlled at the microscale, have been used [46,47]. Such gradient substrates allow for the investigation of critical decision-making machinery required by cells undergoing 2D migration. In most cases, cells have been shown to polarize and migrate in the direction of increasing stiffness. These substrate hold great promise to further explore the role of substrate mechanics in cell movement. As an example, these substrates may prove to be useful in investigating the potential role of the Microtubule Organizing center (MTOC) in the organization and polarization of the cytoskeleton during cell migration [48].

## 3. Moving into the Third Dimension

**Figure 2:**
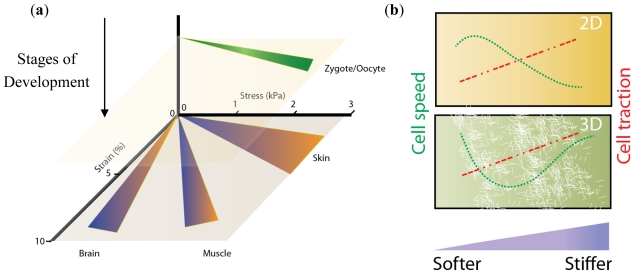
(**a**) Variation of tissue-stiffness with development timeline [52–54]; (**b**) General dependence of cell-speed and traction on stiffness of 2D and 3D substrates [55,56]. Figures not drawn to scale.

The importance of dimensionality in cell culture is gaining prominence due to recent evidence that migration [19], drug responsiveness [49], and differentiation [50] pathways are all altered in 2D *versus* 3D environments [21]. The importance of 3D cell cultures has been illustrated in a number of systems. Most notably, it has been found that during development (Figure 2(a)) there is a marked anisotropy in tissue stiffness that alters differentiation [21]. Additionally, pathogenic conditions like tumorigenesis correlate with altered 3D microenvironments that drive disease [51].

Naturally-derived matrices such as collagen are one of the most often used 3D platforms for the study of cell behavior in *in vivo* like conditions. On a macroscopic level, cells embedded inside a collagen gel are exposed to isotropic conditions. Therefore, the baso-apical polarity which is artificially induced in cells plated on 2D surfaces can be avoided. This is particularly important for studies that involve cells that adhere to a 3D microenvironment *in vivo*, without apical-basal polarizing cues from the ECM.

Embedding cells within a 3D environment imposes an even more critical need to precisely control the physical and chemical properties of the microenvironment. Minor changes in stiffness [57], arrangement of polymerized fibers [58], and ligand presentation [58,59] within the collagen microenvironment have all been shown to affect the reproducibility of results obtained using 3D cell cultures. Additionally, changing the ligand presentation inside collagen gels most often requires a change in the concentration of collagen in the final gel. However, this cannot be achieved without a corresponding change in the stiffness or the pore size of the gel unless the gelation process is modified as discussed in the next section.

### 3.1. Stiffness, Porosity, Ligand-Presentation: The Triad

As described above, there is increasing interest in investigating the role of matrix stiffness on dictating cell behaviors. The stiffness of healthy tissues (as well as of diseased tissues) spans a huge range of values (Table 1). Stiffness of collagen gels, one of the more often used materials for 3D culture, is determined by the spatial density of crosslinks between collagen monomers: the higher the concentration of collagen is in the final gel, the more crosslinking there is and the higher the stiffness is [60]. Collagen I is most often extracted from the collagen-rich tendons in the tails of rats. Traditionally, digestion with acetic acid is preferred over digestion with enzymes, primarily due to generation of non-physiological uncross-linked collagen in the presence of enzymes such as pepsin [61]. When collagen I is extracted with acetic acid and allowed to polymerize, the end-to-end crosslinks formed between individual monomers initiate a chain reaction leading to the formation of collagen fibers arranged in a random orientation [62]. The concentration of collagen I in the final state is often used to regulate the stiffness of the cellular microenvironment. However, owing to direct dependence between the stiffness of collagen architecture, the number of ligands presented to the cell, and the pore size of interstitial spaces, it is difficult to change only one of these parameter without affecting the others.

Of late, several techniques have been proposed to decouple stiffness, porosity and ligand presentation. For instance, stiffness of collagen gels can be manipulated through non-enzymatic glycation [62]. Such an approach can increase gel stiffness while keeping the ligand presentation the same. Glycation has been used in the field of tissue engineering to stiffen constructs over time using sugars. This process can be slow, occurring over several weeks, because the concentrations of sugars are kept low to avoid large changes in osmolarity that could result in cell death. Methods to glycate the collagen prior to polymerization have recently been described [62]. This approach offers the advantage that, because cells are not present at the time of glycation, higher concentrations of sugars can be used and glycation takes less time. A drawback of this approach is that the pore and fiber size may change depending on the extent of glycation. Further work in this regard is expected to decouple all three of these critical characteristics of the matrix.

The pore size of the cellular microenvironment is critical for both the transport of macromolecules and the dynamics of protrusions emanating from the cell membrane. One of the key motivations behind the control of pore size for cell migration studies is that creating well-defined, controlled pores may help distinguish between successful, motility-inducing functional protrusions from protrusions that are unable to lend motility to the cell. The effectiveness of such protrusions is largely a function of the robustness of cell-ECM interactions (receptor-ligand interactions). Therefore, maintaining a constant level of ligand-presentation while altering only the pore size or the stiffness of the mesh of collagen fibers (or biomaterial of choice) is necessary to fully understand the process of cell protrusion formation in cell migration in 3D.

While precise control over pore size within a matrix is very difficult to realize, advances in this area have been proposed using supercritical fluid processing methods [63]. In this case, thermal stresses, rather than chemical agents, drive the processes of polymerization. While results using poly(lactic-coglycolic) acid (PLGA) to test this process are very promising [64,65] further work needs to be done in realizing strict control over pore size in collagen gels. More conventional methods of controlling pore size in collagen have also been reported. The most utilized is control over gelation temperature to control architecture [66]. Changes in temperature can alter polymerization dynamics, which alters pore size. However, these methods are not necessarily easily reproducible and even minor unanticipated changes in gelation conditions can unexpectedly change the resulting fiber architecture.

### 3.2. Matrix Tension and Fiber Alignment

Matrix tension can change in a number of diseases, including notably cardiovascular disease and cancer. Recent evidence suggests that the alignment of fibers within a matrix and the extent to which those fibers are under tension can alter cell behavior. Notably, *in vitro* experiments using collagen gels have shown that collagen matrices that are not in mechanical contact with the walls of a vessel (so called “floating” gels) induce different cell proliferation and migration behavior as compared to gels that are firmly attached to the walls [17,67,68]. It can be argued that a “floating” gel (*i.e.*, a gel that is not mechanically bound to external surfaces) has a different distribution of residual strains as compared to gels that are “tethered” to the walls. The response of the matrix to cell-induced tensile forces therefore becomes very important.

Metallo-proteases (MMPs) secreted by cells are responsible for cleavage of ECM fibers, allowing cells to move through and remodel their matrix [69]. While this cleavage itself can alter the local chemical and mechanical properties of a matrix, there is some evidence to suggest that ECM cleavage is dependent on ECM tension. Already, there are contradictory reports that conclude that tension on a collagen I fiber makes the fiber both more *and* less prone to cleavage by metallo-proteases [70,71] (MMPs). While these studies are all *in vitro* using purified MMP’s, the impact of matrix tension on the degree of matrix cleavage *in vivo* needs to be confirmed. It is possible that increased tension enhances cell migration by increasing MMP activity, however further experiments are necessary to investigate the relationship between fiber tension and MMP function.

Analogous to the effects of matrix tension, matrix fiber alignment can also alter cell migration. Experiments using confocal reflection microscopy have shown that the degree of alignment of collagen fibers near the protruding end of a migrating cell increases [14]. Immediately, these results highlight the need for a system where the alignment of fibers can be modulated. Several teams have described methodologies that yield highly aligned collagen structures—from electrospinning of collagen [72] to extrusion methods [60,73,74]. As described earlier, *in vivo* imaging has revealed cells moving at speeds as high as 3 μm/min; it is expected that the faster modes of cell migration may be best explored in these aligned collagen-substrates.

## 4. Bones to Pick: Interfaces between Tissues

**Figure 3:**
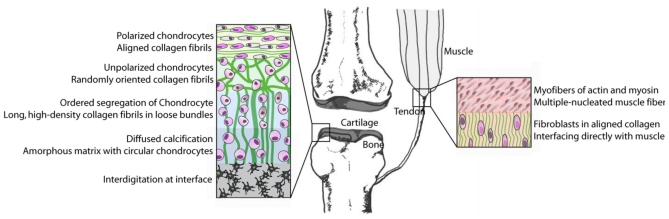
Complexity of tissue-interfaces. Stiffness changes from the order of giga-Pascals [78] to a few kilo-Pascals over relatively short distances. Likewise, degree of calcification, porosity, and microstructure undergo several order of change.

Interfaces between different types of tissues remain the most difficult cellular microenvironment to recreate for both the design of tissue replacements and for the study of cell response to tissue interfaces *in vitro*. Examples of interfaces *in vivo* include the transition from bone to ligament, cartilage to bone, and tendon to bone amongst others. For instance, the transition from a hard osseous tissue to the softer ligaments and finally into very soft connective tissues is accomplished by a gradual change in the cellular and extracellular composition (Figure 3). Interfaces between tissues are often notable for their small length scales, sometimes as small as 100 μm [75,76]. Moreover, interfaces involve heterogeneity of cell types and mineral and protein content. A key concern in artificial generation of interfaces is that the recreated heterogeneity could revert back to homotypic phases over time or after implantation of the artificial interface into the target site [77]. Homeostasis of implanted tissue would necessitate favorable communication between dissimilar cell types, while circumventing cues that initiate homotypic segregation of similar cells.

To achieve the desired level of heterogeneity and gradient in composition for both tissue engineered constructs and *in vitro* experiments, several gradient-generating techniques have been described. One very interesting approach has been the usage of gradient retroviral agents in 3D spacing [76]. In this technique, retroviruses coding for specific genes are embedded inside gels in a gradient and the gels is simultaneously uniformly seeded with fibroblasts. Depending upon the identity of the expressed gene, fibroblasts differentiate into osteoblastic or fibroblastic phenotype. Moreover, since the composition and activity of secreted factors depends on cell phenotype, such a system also creates a gradient in mineralization. Ectopic transplantation of this system *in vivo* is found to exhibit remarkable irreversibility of the biogradient.

Gradients have also been shown to be created using localized alignment of ECM fibers to induce cells to secrete collagen depending on the extent of alignment to which they are exposed [79]. Using electrospinning, it was shown that cells create their own gradient of secreted ECM when placed in a 3D space exhibiting a transition from aligned to random polycaprolactone fibers [73]. A similar approach for other biomaterials could introduce many potential options for activating the intrinsic tissue remodeling machinery of the existing cells.

While electrospinning has been used extensively to achieve strict control over 3D alignment/porosity, extrusion of polymer precursors may be a less harsh tactic that may also offer the opportunity to create interfaces of tissues. Extrusion of collagen I through thin orifices followed by rapid fibrillogenesis can induce a very high degree of alignment in the extruded collagen [74]. Using this system, it has been shown that the aligned collagen can then be used as a 2D substrate, where the alignment of fibroblasts plated on the collagen is found to be very similar to the alignment of collagen, or it could be used as a 3D matrix which encapsulates cells. Due to hydrodynamic nature of extrusion, it may be possible to create a gradient of fiber alignment that could further aid in the development of viable tissue interfaces.

Tissue bonding is another example of an exciting technique being pursued to create physiologically permanent interfaces [80]. Using a thermally-driven gelation system, “integrating fibers” that span the interface between two distinct zones (a zone comprising of preformed collagen fibers and another in liquid phase held at sub-gelation temperature) can be formed. Moreover, it has been found that by controlling the temperature differential between the two zones of collagen gelation, the thickness of the interface can be controlled. The primary advantage of this method is that the generation of the gradient is carried out under a physiologically viable temperature regime, thereby allowing for the generation of interface while cells are still embedded inside the collagen.

## 5. Conclusions

One of the critical challenges to tissue engineering as well as basic cell biology research is the mismatch between *in vitro* cell culture systems and the environment as it exists inside the human body. The behaviors and phenotypes of cells in culture can often be an artifact of an improper or non-physiological cellular microenvironment. Parameters including topography, stiffness, porosity, matrix tension and alignment are all critical determinants of cell function and need to be tuned to achieve experiments which are truly recapitulating what happens *in vivo*. The need for the creation of accurate cell microenvironments has driven the field of biomaterials to develop methods which exact greater control in both real-time tunable systems (for action-as-it-happens experiments) and in systems requiring long-term homeostasis such as tissue engineered implants.

## Figures and Tables

**Table 1:** Variation of tissue stiffness with site and pathogenic state.

Tissue Type	Young’s modulus (kPa)	Reference
**Bovine Aorta**	2.5–2.7	[33]
**Human breast tissue**	0.1–30	[34,35]
**Human breast carcinoma**	4–75	[35,36]
**Human skeletal muscle**	10–75	[37]
**Atherosclerotic lesions in aorta**	10–100	[38]
**Porcine liver**	12–13	[39]
**Prostrate carcinoma**	10–100	[40]
**Cholangiocellular carcinoma**	69–75	[41]
